# Parkin Deficiency Delays Motor Decline and Disease Manifestation in a Mouse Model of Synucleinopathy

**DOI:** 10.1371/journal.pone.0006629

**Published:** 2009-08-14

**Authors:** Margot Fournier, Jérémie Vitte, Jérôme Garrigue, Dominique Langui, Jean-Philippe Dullin, Françoise Saurini, Naïma Hanoun, Fernando Perez-Diaz, Fabien Cornilleau, Chantal Joubert, Héctor Ardila-Osorio, Sabine Traver, René Duchateau, Cécile Goujet-Zalc, Katerina Paleologou, Hilal A. Lashuel, Christian Haass, Charles Duyckaerts, Charles Cohen-Salmon, Philipp J. Kahle, Michel Hamon, Alexis Brice, Olga Corti

**Affiliations:** 1 Université Pierre et Marie Curie-Paris 6, CRICM (Centre de Recherche de l′Institut du Cerveau et de la Moelle épinière), UMR-S975, Paris, France; 2 Inserm (Institut National de la Santé et de la Recherche Médicale), U975, Paris, France; 3 CNRS (Centre National de la Recherche Scientifique), UMR 7225, Paris, France; 4 Université Pierre et Marie Curie-Paris 6, CRPN (Centre de Recherche en Psychiatrie et Neurosciences), UMR-S894, Paris, France; 5 Inserm, U894, Paris, France; 6 CNRS, SEAT (Service d'Expérimentation Animale et de Transgenèse), Villejuif, France; 7 Laboratory of Molecular Neurobiology and Neuroproteomics, EPFL (Ecole Polytechnique Fédérale de Lausanne), Lausanne, Switzerland; 8 Department of Biochemistry, Adolf-Butenandt-Institut, Ludwig-Maximilians University, Munich, Germany; 9 Inserm, U676, Paris, France; 10 Department of Neurodegeneration, Hertie Institute for Clinical Brain Research, Tübingen, Germany; 11 Department of Genetics and Cytogenetics, AP-HP (Assistance Publique Hôpitaux de Paris), Groupe Hospitalier Pitié-Salpêtrière, Paris, France; National Institutes of Health, United States of America

## Abstract

In synucleinopathies, including Parkinson's disease, partially ubiquitylated α-synuclein species phosphorylated on serine 129 (P^S129^-α-synuclein) accumulate abnormally. Parkin, an ubiquitin-protein ligase that is dysfunctional in autosomal recessive parkinsonism, protects against α-synuclein-mediated toxicity in various models.

We analyzed the effects of Parkin deficiency in a mouse model of synucleinopathy to explore the possibility that Parkin and α-synuclein act in the same biochemical pathway. Whether or not Parkin was present, these mice developed an age-dependent neurodegenerative disorder preceded by a progressive decline in performance in tasks predictive of sensorimotor dysfunction. The symptoms were accompanied by the deposition of P^S129^-α-synuclein but not P^S87^-α-synuclein in neuronal cell bodies and neuritic processes throughout the brainstem and the spinal cord; activation of caspase 9 was observed in 5% of the P^S129^-α-synuclein-positive neurons. As in Lewy bodies, ubiquitin-immunoreactivity, albeit less abundant, was invariably co-localized with P^S129^-α-synuclein. During late disease stages, the disease-specific neuropathological features revealed by ubiquitin- and P^S129^-α-synuclein-specific antibodies were similar in mice with or without Parkin. However, the proportion of P^S129^-α-synuclein-immunoreactive neuronal cell bodies and neurites co-stained for ubiquitin was lower in the absence than in the presence of Parkin, suggesting less advanced synucleinopathy. Moreover, sensorimotor impairment and manifestation of the neurodegenerative phenotype due to overproduction of human α-synuclein were significantly delayed in Parkin-deficient mice.

These findings raise the possibility that effective compensatory mechanisms modulate the phenotypic expression of disease in *parkin*-related parkinsonism.

## Introduction

α-Synuclein- and ubiquitin-immunoreactive protein inclusions in neuronal cell bodies and processes (Lewy bodies, LBs; Lewy neurites) or in glial cells are pathological markers of a subset of neurodegenerative diseases referred to as synucleinopathies; these diseases include Parkinson's disease (PD), dementia with LBs and other LB diseases, and multiple system atrophy. Phosphorylation of serine 129 is a widespread α-synuclein modification in LBs, whereas serine 87, found to be constitutively phosphorylated in cell lines, is not modified in these inclusions [1], [2]. A fraction of α-synuclein is covalently modified by one to three ubiquitin molecules in these inclusions [2]–[4]; there is biochemical evidence that α-synuclein phosphorylated on serine 129 (P^S129^-α-synuclein) is the major substrate for ubiquitylation [2]. Three rare missense mutations in the α-synuclein gene, *SCNA*, and more frequently duplications or triplications of genomic regions including *SCNA* are responsible for parkinsonism with autosomal dominant inheritance [5]. The severity of the phenotype correlates with the number of *SCNA* copies in patients with genomic multiplications [6], [7], and supernumerary *SCNA* alleles are associated with increased protein load in both the blood and brain of affected individuals [8]. Thus, the pathogenesis of these familial parkinsonian syndromes appears to be a consequence of the overproduction of normal α-synuclein.

Overproduction of normal or mutated α-synuclein in invertebrate and rodent models leads to synucleinopathy and neurodegeneration [9]–[14]. Mice transgenic for human A30P or A53T α-synuclein develop adult-onset neurodegenerative disorders leading to progressive paralysis due to pathological alterations in brainstem and spinal cord neuronal cell bodies or axons, concomitant with the deposition of α-synuclein- and ubiquitin-immunoreactive material throughout these regions [9], [10], [14], [15]. Despite these striking parallels with human synucleinopathies, the molecular mechanisms underlying α-synuclein toxicity remain unclear. α-Synuclein aggregation *in vitro* is a multi-step process involving the formation of oligomers, protofibrils and insoluble amyloid fibrils [16]. How, and in which order, this process is modulated *in vivo* by phosphorylation, ubiquitylation and possibly other modifications is not known, and there is debate concerning the contributions of the various molecular species to neurodegeneration.

Parkin is an E3 ubiquitin-protein ligase that is abnormal in many cases of autosomal recessive parkinsonism [17]. Carriers of *parkin* mutations have a more favourable disease course compared to sporadic PD patients: although onset is generally earlier, disease progression is significantly slower and the response to dopaminergic treatment better than in sporadic PD [18], [19]. Loss of Parkin function, due to disease-causing *parkin* mutations, may impair proteasomal degradation of toxic substrates, but may also compromise non-proteolytic ubiquitin-dependent pathways related to protein aggregation, protein sorting or cell signalling [20]–[26]; partial loss of Parkin function, through age-related or pathological alterations in its biochemical properties, may also contribute to neurodegeneration associated with sporadic PD and possibly other synucleinopathies [27]–[29]. Whether α-synuclein plays a role in *parkin*-related parkinsonism is unclear. Of eight PD cases with homozygous or compound heterozygous *parkin* mutations analyzed *post mortem*, only three had typical LBs and even here in small numbers, and only one had basophilic α-synuclein-immunopositive dendritic inclusions [30]. Although native α-synuclein is not a Parkin substrate [31], overproduction of Parkin provided protection in a variety of models associated with the short-term accumulation of α-synuclein, suggesting that these proteins are functionally linked [32]–[35]. We explored this possibility further, by evaluating the effects of Parkin deficiency in transgenic mice producing human A30P α-synuclein under the control of the Thy1 promoter (hA30Pα-syn mice). These mice develop α-synuclein-dependent neurodegeneration over a mean period of twenty months. Surprisingly, Parkin deficiency significantly retarded disease manifestation in this model. These findings suggest that loss of Parkin function may act as a beneficial modifier of α-synucleinopathy in slowly progressing *parkin*-related parkinsonian syndromes.

## Results

### Parkin-deficiency delays progression of the neurodegenerative phenotype in transgenic hA30Pα-syn mice

**Figure 1:**
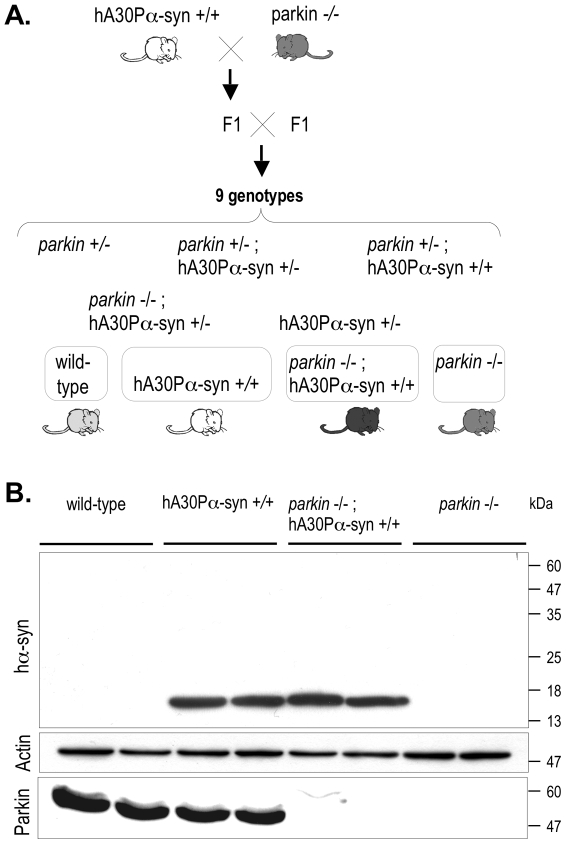
Generation of mice overproducing human α-synuclein in a Parkin-deficient background. (A) Breeding strategy. Parental homozygous *parkin −/−* and hA30Pα-syn +/+ mice were intercrossed. The breeding of the double heterozygous mice (F1) led to the generation of littermates of the nine expected genotypes, four of which, highlighted by the surrounding frames, were used for subsequent analyses. (B) Representative western blot analysis of brain extracts of 17 months-old mice showing the expression of the endogenous *parkin* gene and of the hA30Pα-syn transgene, normalized to actin levels.

A mouse model transgenic for human A30P α-synuclein and deficient for Parkin was generated through a breeding strategy aimed at minimising strain effects, using two previously established mouse lines: *parkin* exon 3-deleted mice (*parkin −/−*) [36] and hA30Pα-syn mice [14], [37] (Figure 1A). Homozygous *parkin −/−* mice were bred with homozygous hA30Pα-syn mice. The double heterozygous generation was intercrossed to generate littermates of the nine expected genotypes in proportions consistent with Mendelian inheritance. Double homozygous mice and their age-matched littermates of wild-type or single homozygous, parental genotypes were kept for subsequent analyses. Appropriate expression of the endogenous *parkin* gene and the α-synuclein transgene were confirmed by western blotting with whole brain extracts (Figure 1B).

**Figure 2:**
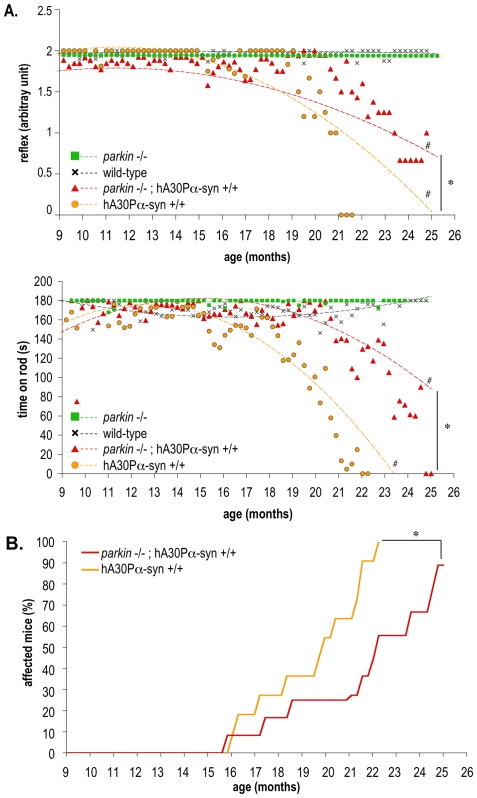
Progression of the neurodegenerative phenotype is delayed by Parkin deficiency in hA30Pα-syn mice. (A) Motor function was assessed by evaluating the hindlimb extension reflex (upper graph) and rotarod performance (lower graph). Mean performance is plotted against age for each genotype and the curvature and slope of parabolic regressions were analysed statistically (n = 10–16). #, slope and curvature different from wild-type and *parkin* −/− with *p*<0.0001; *, different curvatures with *p*<0.05. (B) Age-dependent penetrance of the neurodegenerative phenotype in hA30Pα-syn mice with or without Parkin (n = 12–14). *, *p*<0.01 (Kaplan-Meier analysis).

As expected, adult transgenic hA30Pα-syn mice with or without Parkin developed a neurodegenerative phenotype with age-dependent penetrance, characterized by rapidly deteriorating motor performance, resulting in paralysis of the hindlimbs within approximately one month of the appearance of the first observable clinical signs (unsteady gait, hindlimb weakness and dragging) [14]. These symptoms developed in the absence of degeneration of the nigral dopaminergic neurons [14] (Results S1, Figure S2 and Table S1), a neuronal population in which human α-synuclein was not detected by immunohistochemistry (Results S1, Figure S1B). We investigated whether Parkin deficiency affected the incidence and the progression of this neurodegenerative phenotype: hA30Pα-syn mice with or without a functional *parkin* gene and their littermates of wild-type and parental genotypes were followed from 9 months of age until death to unravel potential disease-predictive changes in motor function (Figure 2). Every week, we tested the hindlimb extension reflex, which is altered by motor neuron diseases [38], and performance on a rotarod, which is progressively impaired in hA30Pα-syn mice [39]. The mean performance in each task was plotted against age for each genotype and the trends were analysed using a second order polynomial curve fit (Figure 2A). The mean performance of wild-type and *parkin* −/− mice in both tasks was similar and remained constant throughout the testing period; in contrast, a progressive, disease-predictive decline in performance was observed in hA30Pα-syn mice. In addition, the decline in performance of both tasks was significantly delayed in the absence of Parkin (Figure 2A).

Consistent with delayed motor impairment in *parkin* −/− mice, the manifestation of the neurodegenerative disorder caused by overproduction of human α-synuclein – here defined as the inability to perform the rotarod task (performance = 0) – was significantly retarded in the absence of Parkin, as illustrated by Kaplan-Meier curves representing the age-dependent penetrance of disease in hA30Pα-syn mice (Figure 2B).

### Accumulation of ubiquitin and P^S129^-α-synuclein but not P^S87^-α-synuclein is associated with disease in hA30Pα-syn transgenic mice

**Figure 3:**
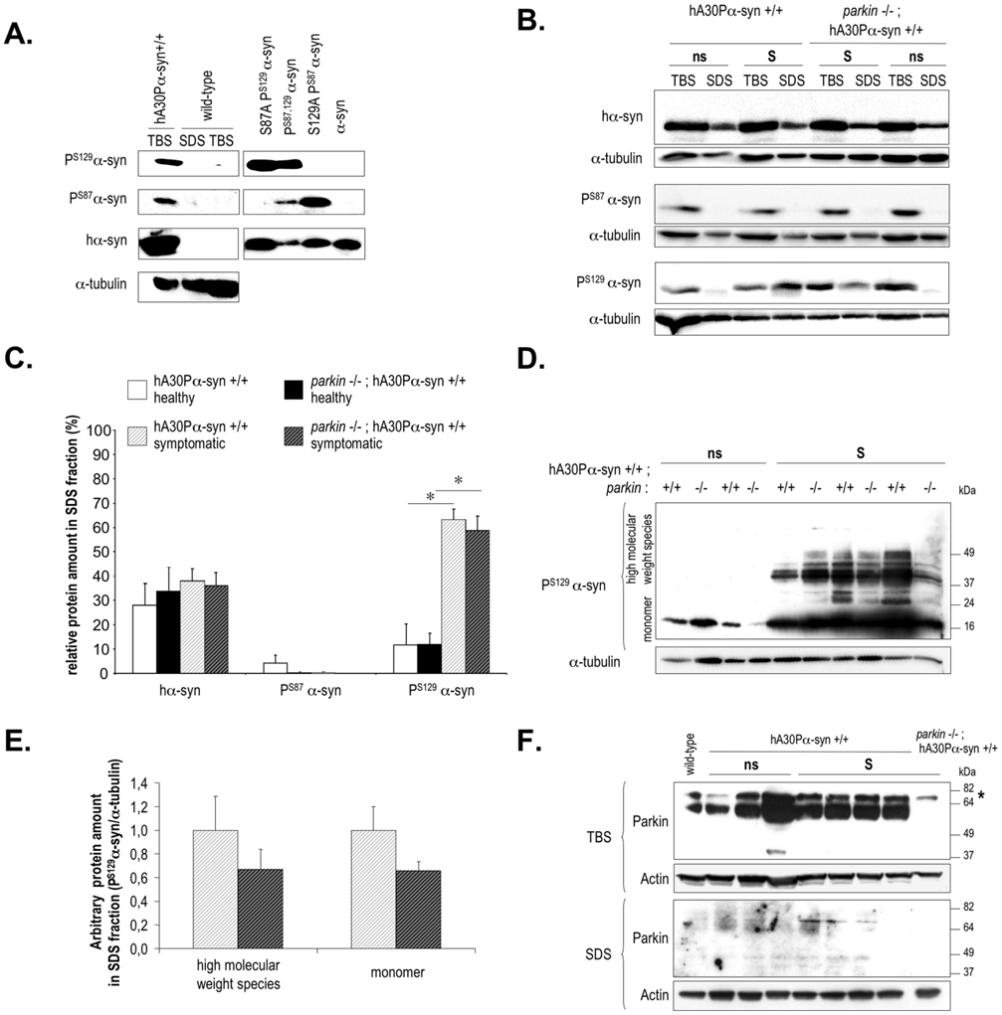
The solubility of P^S129^-α-synuclein but not P^S87^-α-synuclein species is decreased in the brains of end-stage symptomatic hA30Pα-syn mice. (A) Representative western blot using protein-specific antibodies illustrating recognition of human α-synuclein and its isoforms phosphorylated at serine 87 or 129, in the TBS-soluble brain fraction of a symptomatic transgenic hA30Pα-syn mouse. These proteins were not detected in the TBS- or SDS-soluble brain fractions of an aged wild-type control animal (left panel). The anti-P^S87^- and anti-P^S129^-α-synuclein antibodies detected specifically normal recombinant α-synuclein (P^S87,129^α-syn), phosphorylated *in vitro* by casein kinase 1 as described previously [42], but not the non phosphorylated protein (α-syn). As expected, the anti-P^S87^-α-synuclein antibody also recognized the *in vitro*-phosphorylated S129A α-synuclein variant (S129A P^S87^α-syn), but not phosphorylated S87A α-synuclein (S87A P^S129^α-syn). In contrast, the anti-P^S129^-α-synuclein antibody recognized S87A P^S129^α-syn but not S129A P^S87^α-syn. (B) Representative western blots illustrating α-synuclein (top panel), P^S87^-α-synuclein and P^S129^-α-synuclein (middle and bottom panels) protein abundance in TBS-soluble and -insoluble (SDS) brain fractions from symptomatic (S) and non symptomatic (ns) mice with and without Parkin. (C) Quantitative analysis of human α-synuclein, P^S87^-α-synuclein and P^S129^-α-synuclein solubility in symptomatic and non symptomatic hA30Pα-syn mice with (ns: n = 4; S: n = 13−8) and without (ns: n = 4; S: n = 11−8) Parkin. The relative protein amount was defined as the ratio between the amount of protein normalized to α-tubulin in the SDS fraction, and the total amount of protein normalized to -tubulin in the SDS and TBS fractions. The abundance of P^S129^-α-synuclein in the SDS brain fraction was significantly higher in symptomatic mice in the presence or absence of Parkin than in healthy mice. *, *p*<0.01. (D) Representative western blots showing P^S129^-α-synuclein immunoreactive species with retarded electrophoretic mobility after long exposure times in the SDS fraction from symptomatic mice. (E) Quantitative analysis of the monomer (short exposure times) and the P^S129^-α-synuclein species with higher apparent molecular mass (long exposure times) in symptomatic mice with (n = 13) and without (n = 11) Parkin. (F) Western blot analysis with anti-Parkin antibodies of TBS and SDS fractions from a wild-type mouse, a representative pool of healthy and symptomatic hA30Pα-syn mice, and a symptomatic Parkin-deficient hA30Pα-syn control mouse. Parkin was abundant in the TBS fractions but was not detected in the SDS fractions from healthy or symptomatic mice, even after long exposure times. The asterisk indicates a non specific band recognized by anti-Parkin antibodies.

We assayed total human α-synuclein, P^S129^-α-synuclein and P^S87^-α-synuclein in aged healthy and end-stage symptomatic hA30Pα-syn mice with and without Parkin with antibodies recognizing specifically each of these proteins (Figure 3A). Total human α-synuclein was found in the buffer-soluble (TBS) and -insoluble (SDS) brain fractions (Figure 3B, upper panel); it was for the most part soluble and its relative solubility did not change with the appearance of symptoms and was similar in the presence and absence of Parkin (Figure 3C). P^S87^-α-synuclein was only found in the TBS fraction, and the amounts found were similar for healthy and symptomatic mice with or without Parkin (Figure 3B, middle panel, Figure 3C). Most P^S129^-α-synuclein in healthy mice was soluble, with approximately 10% of the protein being buffer-insoluble, both in the presence and absence of Parkin (Figure 3B, lower panel, Figure 3C). In contrast, insoluble P^S129^-α-synuclein species accumulated in symptomatic mice, with approximately 60% of the protein found in the SDS fraction whether Parkin was present or not (p<0.001 between symptomatic and non symptomatic mice). In this fraction, long exposure times revealed variable amounts of P^S129^-α-synuclein species with retarded electrophoretic mobilities only in symptomatic mice (Figure 3D and E). Soluble Parkin was readily detected in the TBS fractions from healthy and symptomatic hA30Pα-syn mice with a functional *parkin* gene. In contrast, it was not observed in the SDS fractions even after long exposure times (Figure 3F).

**Figure 4:**
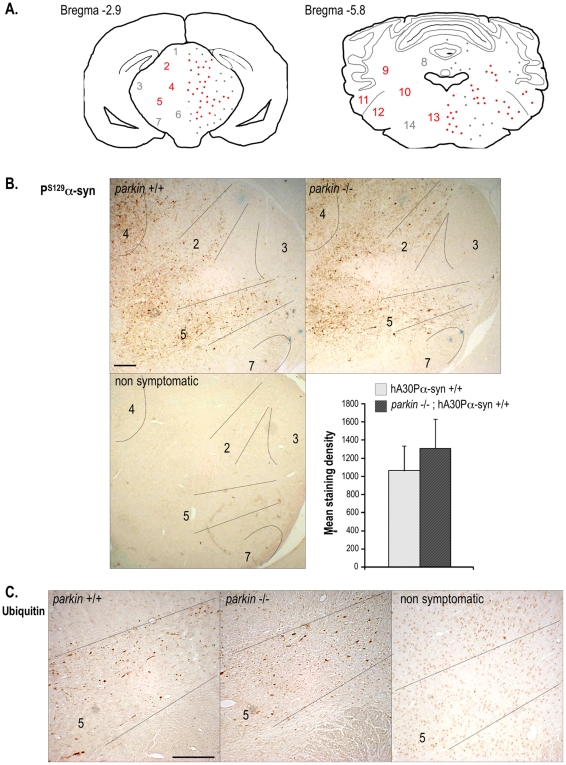
Distribution of P^S129^-α-synuclein- and ubiquitin-immunolabelling in symptomatic hA30Pα-syn mice. (A) Schematic illustration of the distribution and density of P^S129^-α-synuclein deposits on representative brain coronal sections of symptomatic mice. Similar distributions were observed in hA30Pα-syn mice with or without Parkin. Regions immunolabelled in most animals are indicated in red, whereas those less frequently stained are in grey. (B) Representative micrographs illustrating P^S129^-α-synuclein-immunolabelling in the brainstem (bregma −2.9) of symptomatic hA30Pα-syn mice with and without Parkin, and in a healthy hA30Pα-syn control mouse. Quantitative analysis of the density of P^S129^-α-synuclein-labelling in a representative region of the brainstem of symptomatic mice (n = 5 per genotype). (C) Representative micrographs illustrating typical ubiquitin-immunoreactivity in the presence or absence of Parkin in the brainstem of symptomatic mice, and of a healthy hA30Pα-syn control mouse. 1- *superior colliculus*, 2- pretectal nuclei, 3- geniculate nuclei, 4- periaqueductal grey layer, 5- *zona incerta*, 6- ventral tegmental area, 7- SN *pars reticulata*, 8- cerebellar white layer, 9- lateral cerebellar nuclei, 10- vestibular nuclei, 11- cochlear nuclei 12- trigeminal nuclei, 13- pontine reticular nuclei, 14- parvicellular reticular nuclei. Scale bars indicate 200 µm.

**Figure 5:**
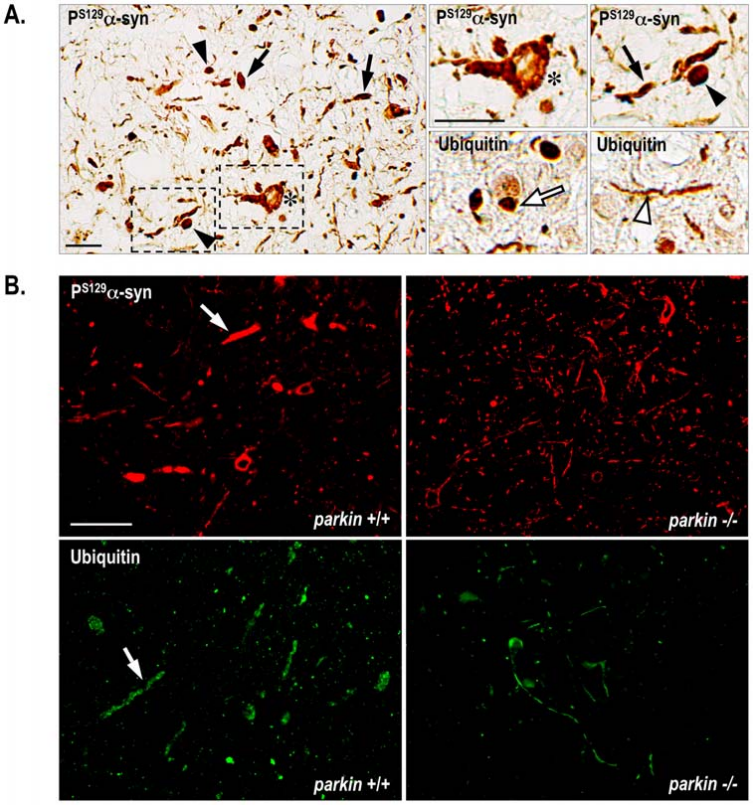
Parkin deficiency mitigates neuritic pathology in end-stage symptomatic hA30Pα-syn mice. (A) Representative micrographs illustrating P^S129^-α-synuclein and ubiquitin immunoreactivities in the brainstem of hA30Pα-syn symptomatic mice. Swollen, spherical (black arrowheads) and elongated (black arrows) neuritic profiles, thin, thread-like neurites (white arrowhead), rare compact inclusions (white arrow), and immunostaining throughout the somatodendritic compartment (stars) were observed. The two upper right micrographs correspond to a higher magnification of the regions outlined by the frames in the left micrograph. Scale bars indicate 20 µm. (B) Representative micrographs illustrating P^S129^-α-synuclein and ubiquitin immunoreactivities in the grey matter of the cervical spinal cord from hA30Pα-syn mice with (left column) and without Parkin (right column). Note the abundance of aberrantly swollen neuritic processes (white arrows) in the presence of Parkin. Scale bar indicates 60 µm.

We used immunohistochemistry to study the regional distribution of total human α-synuclein, P^S129^-α-synuclein, P^S87^-α-synuclein and ubiquitin in the central nervous system of aged healthy and end-stage symptomatic mice with and without Parkin. Similar distributions were found for total human α-synuclein in the presence and absence of Parkin, whether the animals had symptoms or not (Results S1, Figure S1A). P^S87^-α-synuclein immunoreactivity was undetectable in any of the conditions studied (data not shown). In contrast, there was extensive P^S129^-α-synuclein immunoreactivity in various regions of the brainstem and the spinal cord in end-stage symptomatic hA30Pα-syn mice with or without Parkin, but not in their non symptomatic littermates (Figure 4A, B and Figure 5A, B). The extent of the P^S129^-α-synuclein pathology was variable between animals but was similar in the presence or absence of Parkin (Figure 4B). End-stage symptomatic hA30Pα-syn mice but not their healthy littermates also displayed variable severity of ubiquitin pathology (Figures 4C and 5B). The regional distribution of ubiquitin overlapped with that of P^S129^-α-synuclein, but was generally less extensive; it was observed regardless of the *parkin* genotype.

### Parkin deficiency mitigates ubiquitin pathology in hA30Pα-syn transgenic mice

Staining for P^S129^-α-synuclein and for ubiquitin in end-stage symptomatic hA30Pα-syn mice with and without Parkin appeared in thin, tortuous, often short neuritic extensions; in hypertrophied, spherical or elongated neuritic profiles; throughout the somatodendritic compartment; and, only rarely, in compact cytoplasmic, nuclear or neuritic inclusions (Figure 5A). The relative frequencies of these qualitatively different categories of labelled structures differed between animals, but they were observed in mice of both genotypes at disease end-stage; in the presence of Parkin, aberrantly swollen neuritic profiles filled with P^S129^-α-synuclein- and ubiquitin-immunoreactive material tended to be more abundant that in its absence, as illustrated on spinal cord sections from representative animals (Figure 5B).

**Figure 6:**
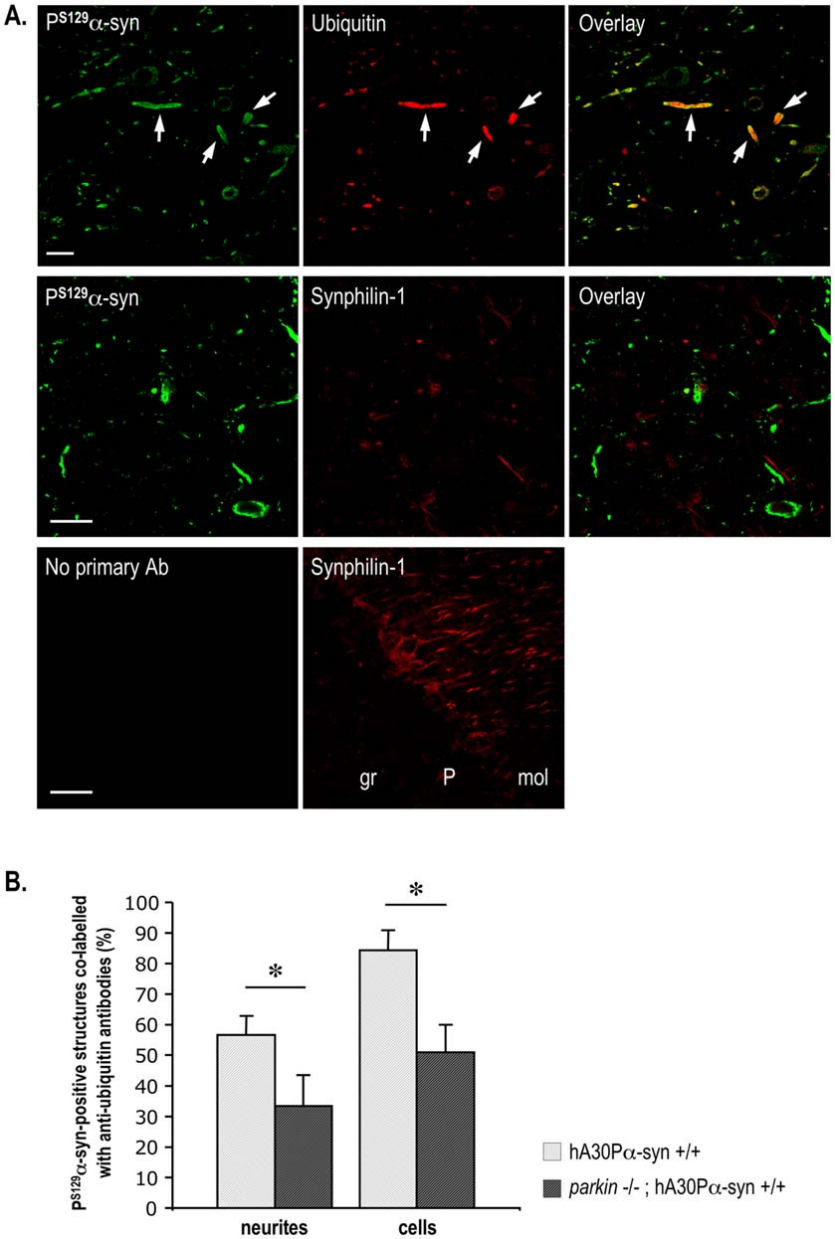
The frequency of P^S129^-α-synuclein-positive structures immunolabelled with anti-ubiquitin antibodies is lower in the absence than presence of Parkin. (A) Confocal scanning laser micrographs of a brainstem section from a representative symptomatic hA30Pα-syn mouse illustrating colocalization of ubiquitin staining with P^S129^-α-synuclein-positive deposits (top panel). Note that ubiquitin was present in most of the swollen P^S129^-α-synuclein-positive neurites (arrows). Synphilin-1-immunoreactivity was not found within the P^S129^-α-synuclein-positive deposits (middle panel), whereas it was detected in nerve processes in a representative region of the cerebellum (lower panel). gr: granular cell layer; P: Purkinje cell layer; mol: molecular cell layer. Scale bars indicate 25 µm. (B) Percentage of P^S129^-α-synuclein-immunopositive neurites and cell bodies co-labelled for ubiquitin in a representative region of the brainstem of symptomatic hA30Pα-syn mice with or without Parkin (n = 4–5). *, *p*<0.05.

We determined whether the ubiquitin staining observed in diseased hA30Pα-syn mice was associated with the deposited P^S129^-α-synuclein species by double fluorescent immunolabelling of representative brain and spinal cord sections and confocal microscopy (Figure 6A, top panel). Most of the ubiquitin staining co-localized with P^S129^-α-synuclein staining (Pearson's colocalization coefficient = 0.8 in the presence and absence of Parkin). Approximately 85% of the cell bodies and 55% of the neurites immunopositive for P^S129^-α-synuclein were co-labelled with anti-ubiquitin antibodies in transgenic hA30Pα-syn mice with Parkin (Figure 6B); in mice deficient for Parkin, only approximately 50% of the cell bodies and 35% of the neurites containing P^S129^-α-synuclein immunopositive material were also ubiquitin-positive. However, more than 90% of the pathologically swollen P^S129^-α-synuclein-positive neurites were co-labelled for ubiquitin, whether Parkin was present or not (Figure 6A, top panel). Immunoreactivity to synphilin-1, a Parkin substrate reported to co-localize with P^S129^-α-synuclein-positive inclusions when overproduced together with α-synuclein in cell models [20], was not associated with P^S129^-α-synuclein-positive protein deposits (Figure 6A, middle and bottom panels).

**Figure 7:**
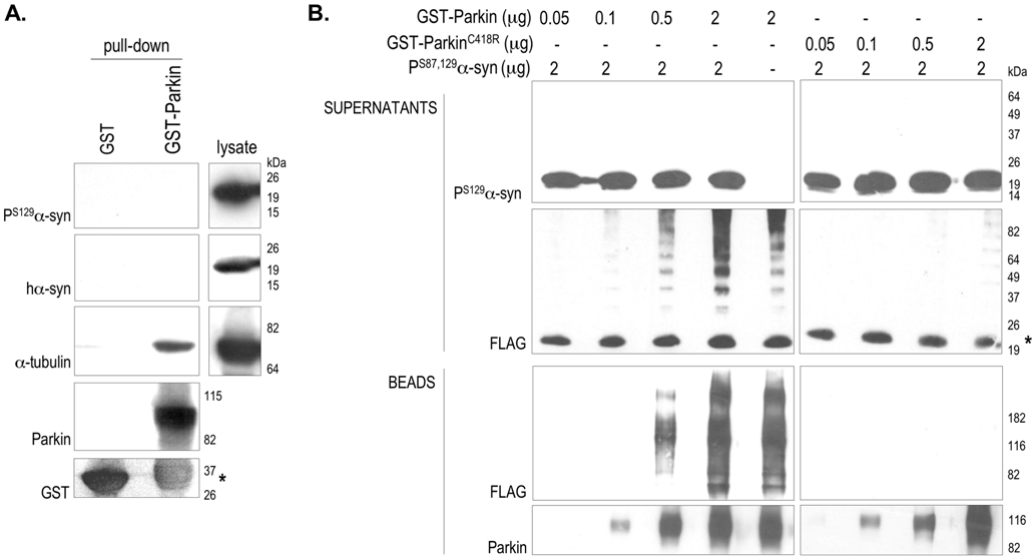
Phosphorylated α-synuclein is not a Parkin substrate. Analysis of a representative GST-pull-down assay performed by incubating GST-Parkin [26] or GST with the TBS-soluble protein fraction from a symptomatic hA30Pα-syn mouse. Antibodies against human α-synuclein, P^S129^-α-synuclein or α-tubulin were used for western blotting. GST-Parkin interacts specifically with α-tubulin [54], but not with α-synuclein or P^S129^-α-synuclein. The asterisk indicates the GST-moiety resulting from the degradation of the proteolytically unstable recombinant GST-Parkin protein. (B) Results of an *in vitro* ubiquitylation assay performed by incubating GST-Parkin, or the enzymatically inactive GST-Parkin^C418R^ variant, with ubiquitin-activating enzyme, ubiquitin-conjugating enzyme, UbcH7, FLAG-tagged ubiquitin and ATP, in the presence or absence of *in vitro*-phosphorylated α-synuclein (P^S87,129^-α-syn). Once the reaction was completed, GST-Parkin was immobilized on glutathione-sepharose beads; the beads and the supernatant fraction were analyzed by western blotting with anti-FLAG, P^S129^-α-synuclein, or anti-Parkin antibodies. GST-Parkin (but not GST-Parkin^C418R^) promoted its own ubiquitylation in a dose-dependent manner, as demonstrated by the amounts of ubiquitylated species in the bead fraction being proportional to the amounts of GST-Parkin used for the ubiquitylation reactions. In addition, GST-Parkin (but not GST-Parkin^C418R^) promoted the formation of ubiquitin chains, as indicated by the presence of a ladder of regularly spaced ubiquitin-immunoreactive protein bands in the supernatant fraction. However, GST-Parkin did not promote the ubiquitylation of P^S129^-α-synuclein: P^S129^-α-synuclein-immunoreactive proteins with higher apparent molecular mass than the monomer were not detected in the supernatant fraction at any of the GST-Parkin concentrations used in the assay (P^S129^-α-syn); moreover, we did not observe any ubiquitin-immunoreactive bands specific for P^S129^-α-synuclein in the supernatant fraction (FLAG). The asterisk indicates a FLAG immunoreactive band most likely corresponding to a thioester adduct formed between the E2 enzyme UbcH7 and FLAG-tagged ubiquitin in the presence of the E1 enzyme.

We used GST pull down and *in vitro* ubiquitylation assays to test whether Parkin interacts directly with and ubiquitylates phosphorylated α-synuclein. GST-Parkin interacted with the Parkin substrate, α-tubulin, but not with P^S129^-α-synuclein when added to brain extracts from symptomatic hA30Pα-syn mice (Figure 7A). In addition, GST-Parkin was unable to promote the ubiquitylation of recombinant, phosphorylated human α-synuclein in a purely *in vitro* ubiquitylation assay (Figure 7B).

### P^S129^α-synuclein-immunoreactivity is associated with caspase 9 activation in hA30Pα-syn transgenic mice

**Figure 8:**
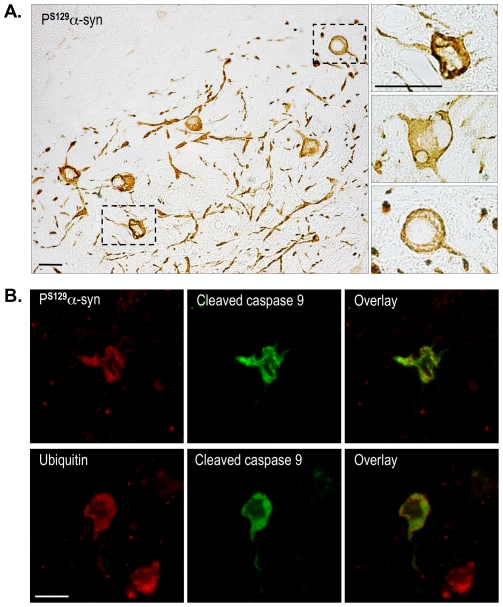
P^S129^-α-synuclein-immunoreactivity is associated with activation of caspase 9. (A) Examples of P^S129^-α-synuclein-positive neurons with abnormally shaped nuclei and dysmorphic or abnormally swollen somata and proximal dendrites in the brainstem of symptomatic hA30Pα-syn mice. The upper and lower right micrographs correspond to a higher magnification of the regions outlined by the frames in the left micrograph. (B) Double immunofluorescent labelling showing caspase 9 activation in representative P^S129^-α-synuclein- or ubiquitin-positive neurons of the spinal cord. Scale bars indicate 30 µm in (A) and 15 µm in (B).

Whether there is a causative relationship between P^S129^-α-synuclein accumulation and the pathogenesis of synucleinopathies is currently a matter of debate [40], [41]–[44]. In diseased hA30Pα-syn mice, many P^S129^-α-synuclein-positive neuronal cell bodies were abnormally shaped, with dysmorphic nuclei or aberrantly swollen cytoplasm and proximal neurites, whether or not they accumulated ubiquitin (Figure 8A). Cleaved capase 9 immunoreactivity has been reported in viral vector-based models of synucleinopathy [44], [45], so we evaluated activation of caspase 9 in end-stage hA30Pα-syn mice (Figure 8B). Cleaved caspase 9 was found in approximately 5% of the neuronal cell bodies accumulating P^S129^-α-synuclein in the spinal cords of transgenic mice (7.4±1.8% with and 4.5%±2.2 without Parkin; p = 0.4), and in a similar proportion of cell bodies of neurons accumulating both P^S129^-α-synuclein and ubiquitin (6.3%±2.8 in the presence of Parkin, 6.7%±2.9 in its absence; p = 0.9). Importantly, deposits of P^S129^-α-synuclein were present in more than 90% of the neurons in which caspase 9 was activated in hA30Pα-syn mice both with (92.0%±3.2) and without Parkin (96.0%±3.1; p = 0. 4).

## Discussion

We report an investigation of the effects of Parkin deficiency on the pathological accumulation of the A30P variant of human α-synuclein and the manifestation of the neurodegenerative phenotype in a transgenic mouse model of synucleinopathy (hA30Pα-syn mice) [14]. Disease onset in hA30Pα-syn mice was preceded by specific, progressive impairment of performance in two tasks —hindlimb extension reflex and rotarod tests— predictive of neuronal dysfunction in sensorimotor nuclei of the brainstem and the spinal cord. Disease progression was accompanied by the specific deposition of insoluble P^S129^-α-synuclein species, detected by biochemical and immunohistochemical methods throughout the brainstem and the spinal cord. In contrast, P^S87^-α-synuclein immunoreactive species readily detected in the soluble brain fractions were not deposited as insoluble material in diseased hA30Pα-syn mice. Previous reports failed to identify P^S87^-α-synuclein-immunoreactive species by biochemical or immunohistochemical techniques in mouse models overproducing human α-synuclein and in brains from synucleinopathy patients, suggesting that P^S87^-α-synuclein is not involved in the physiopathology of neurodegenerative disorders associated with α-synuclein accumulation [2], [46]. Whether there is a causative relationship between P^Ser129^-α-synuclein accumulation and the pathogenesis of synucleinopathies is a matter of controversy which still needs to be clarified [40], [41], [43], [44]. In our model, immunoreactivity to active caspase 9 was observed in 5–10% of the P^S129^-α-synuclein-positive neuronal cell bodies of the spinal cord, and was almost exclusively associated with the presence of deposited P^S129^-α-synuclein, indicating that accumulation of this protein may be a deleterious event related to neurodegeneration. As in human synucleinopathies [2]–[4], [47], some of the P^S129^-α-synuclein deposits were associated with ubiquitin, suggesting that ubiquitylation is a secondary event following protein deposition.

In late-stage symptomatic mice, the disease-specific neuropathological features were similar in the presence and absence of Parkin. However, the proportion of P^S129^-α-synuclein-immunoreactive deposits associated with ubiquitin was lower, indicating less advanced α-synucleinopathy. Moreover, in the absence of Parkin, the mean decline in sensorimotor performance and the manifestation of disease were significantly delayed in hA30Pα-syn mice. These results are in apparent contradiction with previous studies reporting that Parkin protects against α-synuclein-mediated toxicity in primary neurons, in Drosophila and in rats [32]–[35]. However, Parkin was overproduced beyond physiological levels in these models. In addition, cell survival was only monitored in the short-term, raising the possibility that Parkin modulate synucleinopathy with distinct short-term and long-term effects on neuronal viability. When overproduced in the rat *substantia nigra* together with A30P α-synuclein by lentiviral vector-mediated gene transfer, Parkin protected dopaminergic neurons from degeneration six weeks after inoculation of the vectors [35]. Neuroprotection was accompanied by an increase in the proportion of neurons carrying P^S129^-α-synuclein-positive aggregates, suggesting that Parkin promotes the deposition of this protein. Other studies have shown that overproduced Parkin promotes the formation of ubiquitylated aggregates, providing in some cases protection against the toxicity of the involved proteins, perhaps by sequestering diffusible toxic species [20], [24] [48]; in the long term, this process could contribute to neuronal dysfunction and accelerate neurodegeneration. However, Parkin did not bind to P^S129^-α-synuclein in brain extracts from hA30Pα-syn mice, and it was unable to promote the ubiquitylation of phosphorylated α-synuclein *in vitro*. It is therefore unlikely that endogenous Parkin contributes to the deposition of P^S129^-α-synuclein through direct mechanisms.

Compensatory mechanisms triggered by Parkin deficiency may intervene indirectly in delaying synucleinopathy and neurodegeneration in hA30Pα-syn mice. Elevated levels of reduced glutathione have been reported throughout the brain of Parkin-deficient mice. Interestingly, overexpression of genes involved in glutathione biosynthesis and conjugation have each been recently shown to protect against neurodegeneration in a Drosophila model of synucleinopathy [49]. Although the link between increased glutathione levels and decreased α-synuclein toxicity in this model remains to be established, it is plausible that increased antioxidant activities associated with the established role of glutathione in protection against oxidative stress [50] affect α-synuclein aggregation. It would be valuable to determine the relative contribution of *parkin* to the molecular pathway leading to accumulation of α-synuclein and neurodegeneration in hA30Pα-syn mice. Current studies in our laboratory aim at evaluating whether and to what extent the deletion of a single *parkin* allele affects the delay in appearance of signs of motor dysfunction and disease manifestation in this model. These studies should provide useful clues to the mechanisms underlying our current observations; gene dosage-dependent effects would be indicative of direct genetic interaction between *parkin* and *SCNA*, whereas their absence would rather hint at compensatory neuroprotective mechanisms triggered by complete but not partial Parkin depletion.

The effects of Parkin deficiency has been studied in a transgenic mouse model overproducing the A53T variant of human α-synuclein. These mice displayed a neurodegenerative phenotype similar to that observed in hA30Pα-syn mice, but with more severe progression: by 12 months of age, 50% of the hA53Tα-syn mice were affected [51], whereas at this age, none of the hA30Pα-syn mice in our study presented features of disease. As in our model, the absence of Parkin did not affect the amount or distribution of native α-synuclein, or the appearance of ubiquitin-positive profiles; unfortunately, P^S129^-α-synuclein accumulation was not studied. Although a longitudinal analysis of motor performance in hA53Tα-syn mice was not provided, the survival curves with and without Parkin were similar. The differences between our results and those described by von Coelln *et al* may indicate that Parkin deficiency affects A53T and A30P α-synucleinopathies differently; Parkin deficiency may have a smaller effect on A53T α-synuclein-induced neurodegeneration due to the higher intrinsic aggregation potential [52] and toxicity of A53T α-synuclein than A30P α-synuclein, leading to a more rapidly progressive neurodegenerative phenotype in mice [15], [51].

Unfortunately, neither hA53Tα-syn [51] nor hA30Pα-syn mice provided evidence for interaction between Parkin deficiency and synucleinopathy in catecholaminergic brainstem neurons (Figure S2, Table S1). This may be due to the weakness of the promoters used to drive transgene expression in these neuronal populations (Results S1, Figure S1B). Therefore, further work with models in which α-synuclein accumulates abundantly in the dopaminergic neurons of the *substantia nigra*, e.g. through viral vector-mediated gene transfer, is required to confirm our findings.

In conclusion, we provide unprecedented evidence that Parkin deficiency modulates the phenotypic expression of disease in a long-term mouse model of synucleinopathy. These findings raise the intriguing possibility that the loss of functional Parkin is a beneficial modifier of α-synuclein build-up in the slowly progressive *parkin*-related parkinsonian syndromes, in which LB pathology is more rarely observed and less severe than in sporadic PD [30].

## Materials and Methods

### Ethics Statement

All experiments involving mice were approved by the Ile de France Regional Ethics Committee for Animal Experiments, N°3 (P3/2006/006).

### Generation of transgenic mice overproducing human A30P α-synuclein in a Parkin-deficient background

Mice transgenic for human A30P α-synuclein and deficient for Parkin were generated by a crossbreeding strategy aimed at minimising potential strain effects, using two previously established mouse lines: *parkin* exon 3-deleted mice (*parkin* −/−) [36], brought into the C57Bl/6j genetic background by an accelerated backcross procedure; and hA30Pα-syn mice (line 31H) [14], [37], brought into a C57Bl/6j background by eight consecutive backcrosses (Figure 1A). Homozygous *parkin −/−* mice were bred with homozygous hA30Pα-syn mice and mice of the double heterozygous generation were then intercrossed. The progeny of this intercross was genotyped by PCR amplification. Genomic DNA was prepared from tail fragments homogenized in lysis buffer (100 mM Tris-HCl, 0.2% SDS, 200 mM NaCl, 1 mM CaCl_2_) and digested with proteinase K (10 mg/ml) overnight at 55°C; the DNA was precipitated with isopropanol (v/v) and rinsed with 70% ethanol. The *parkin* genotype was determined by two parallel PCRs using sets of oligonucleotide primers specific for the *parkin* gene (F_wt_: 5′-TGCTCTGGGGTTCGT C-3′; R_wt_: 5′-TCCACTGGCAGAGTAAATGT-3′) and for the recombinant knock-out allele (F_KO_: 5′-TTGTTTTGCCAAGTTCTAAT-3′; R_KO_: -TCCACTGGCAGAGTAAATGT-3′). PCR amplification was carried out in 25 µl reaction mixes containing approximately 300 ng of genomic DNA, 2 mM MgCl_2_, 0.2 mM dNTPs, 0.4 µM of each oligonucleotide and 1U Taq polymerase (Abscys) in 1X PCR buffer (Abscys). After an initial denaturation step of 3 min at 94°C, PCR conditions were: 35 cycles of 94°C, 30 s; 58°C, 30 s; 72°C, 40 s; followed by a 10 min extension at 72°C. Semi-quantitative real-time PCR was performed with genomic DNA and an ABI Prism 7700SDS instrument, to determine whether the hA30Pα-syn transgene was present in the heterozygous or homozygous state. The oligonucleotide pair used to amplify the human transgene was: F: 5′-AGGGAGCAGGGAGCATTG -3′; R: 5′-CAGGATCCACAGGCATATC-3′. The amplification was carried out in reaction volumes of 25 µl consisting of SYBR Green PCR Universal Master Mix (Applied Biosystems) containing 1 ng of DNA and 0.8 µM of each oligonucleotide. The reactions were incubated for 2 min at 50°C and for 10 min at 95°C, and then amplifications were performed as follows: 40 cycles of 95°C, 10 s; 60°C, 60 s. Values for the hA30Pα-syn transgene were normalized to those obtained in the same amplification conditions for the endogenous glyceraldehyde-3-phosphate dehydrogenase gene with the oligonucleotides F: 5′-GAACATCATCCCTGCATCCA-3′ and R: 5′-CCAGTGAGCTTCCCGTTCA-3′. Western blotting was used to examine the expression of the endogenous *parkin* gene and of the hA30Pα-syn transgene in hemi-brains from four 17 months-old asymptomatic mice per genotype, as described below.

The frequencies of the nine genotypes of the progeny of the double heterozygous intercross were consistent with Mendelian inheritance. Double homozygous mice and their age-matched littermates of wild-type or single-homozygous, parental genotypes were collected for subsequent analyses. After weaning, male and female mice were separated and maintained at a maximum of five littermates per cage, with food and water *ad libitum*, and at constant temperature (21°C) with a 07:00 – 19:00 light cycle.

### Behavioural analyses

The behaviour of age-matched littermate mice (females) of each selected genotype (n = 10–16) was tested every week, from 9 to 24 months of age. To limit sources of variability, including unnecessary stress, the conditions for testing were kept as constant as possible: the mice were hosted and tested in the same room; the same experimenters (MF and JG), blind to the genotype of the animals throughout the testing period, took care of them and assessed their performance weekly on two consecutive days (Tuesday and Wednesday morning); the tasks were performed in the same order ((i) extension reflex, (ii) rotarod); the between-cage order of testing, initially chosen at random, was kept constant throughout the testing period. Since mice of the same litter were grouped into cages, each cage hosted age-matched animals of different genotypes; the within-cage order of testing was random. Each behavioural session was held during the light period, between 9 a.m. and 12 a.m. and included the two following tests:

Rotarod *—* The time spent on a rod (Columbus Instrument) rotating at a speed of 12 rpm was measured. The test was stopped after an arbitrary period of 180 s. Mice falling before the end of this period were tested a second time, and the best trial (longest time) was recorded.

Hindlimb extension reflex *—* Mice were suspended by their tail and the extension reflex of their hindlimbs was observed. The attribution of arbitrary extension scores was adapted from [38] as follows: 2 for normal extension of both hindlimbs; 1.5 for incomplete extension of one hindlimb; 1 for absence of extension of one hindlimb; 0.5 for absence of extension of one hindlimb and incomplete extension of the second limb; 0 for absence of extension of both hindlimbs.

### Western-blot analysis, GST pull-down and *in vitro* ubiquitylation assays

Brain extracts were fractionated as described [53]. Briefly, posterior hemi-brains (from bregma -2; approximately 150 mg of frozen tissue) were homogenized in TBS (Tris-buffered saline) containing protease (Complete protease inhibitor cocktail, Roche Diagnostics) and phosphatase (30 mM NaF, 30 mM β-glycerophosphate, 0.2 mM Na_3_VO_4_, 5 mM Na-pyrophosphate and 30 nM okadaic acid) inhibitors. The samples were sonicated, centrifuged at low speed (1,000×g, 5 min, 4°C) and ultracentrifuged (130,000×g, 1 h, 4°C). The resulting supernatants (the TBS-soluble fractions) were collected. The pellets were extracted twice with 200 µl of TBS containing 5% SDS, and the resulting supernatants were collected and pooled as the TBS-insoluble (SDS-soluble) fraction. Protein concentrations were determined using the bicucullinic acid (BCA) protein assay (Pierce). To confirm genotypes (Figure 1), TBS-soluble brain fractions (10 µg) from non symptomatic 17 months-old mice were analyzed. TBS- and SDS-soluble brain fractions (30 µg) from 17 months-old non symptomatic and 17–25 months-old symptomatic mice were used for assaying α-synuclein and P^S129^-α-synuclein proteins (Figure 7).

GST pull-down assays were done as described previously [26], with 500 µg TBS-soluble brain fraction from a symptomatic mouse, and 5 µg of GST or GST-Parkin immobilized on glutathion-sepharose beads (Amersham Biosciences). Ubiquitylation assays were carried out as described in [26]. Recombinant normal α-synuclein and the S87A and S129A variants were produced, purified and phosphorylated *in vitro* as described previously [42].

Proteins were resolved by SDS-PAGE on 4–12% gradient gels (Invitrogen). The proteins were electrotransferred onto nitrocellulose membranes and probed with: monoclonal anti-Parkin (MAB5512, clone PRK8, Chemicon), anti P^S129^-α-synuclein (clone PSyn#64, WAKO; clone EP1536Y, Abcam), anti-α-tubulin (clone DM 1A, Sigma), anti-FLAG (M2, Sigma); polyclonal anti-α-synuclein (SA3400, Affiniti) and anti-actin (A2066, Sigma), anti-GST (GE Healthcare/Amersham). Proteins were visualized by enhanced chemioluminescence (Pierce) using a Kodak Image Station 4000 MM. The Image J program (http://rsb.info.nih.gov/ij/) was used for signal quantification.

### Immunohistochemistry

Mice were anaesthetized with pentobarbital (130 mg/kg, i.p.; Sigma, St Quentin, France) and perfused transcardially with 4% paraformaldehyde freshly prepared in 0.1 M PBS, pH 7.4. Brains were removed and post-fixed for two hours in the same solution. Left-half brains were dehydrated by four 10 min incubations and two 20 min incubations in ethanol at 35°C, followed by three incubations of 45, 60 and 75 min each in xylene, at 35°C. The tissues were then embedded in paraffin by three incubations of 40, 15 and 15 min each at 65°C (Shandon Excelsior tissue processor from Thermo Scientific) and cut on a microtome into 5 µm-thick sections collected on slides and stored at room temperature. For immunohistochemistry, sections were deparaffinized in xylene, and hydrated in a descending series of ethanol concentrations. Antigens were retrieved by boiling the sections in 0.01 M citrate buffer, pH 6.0, five times for 3 min in a microwave at 350 Watts.

For immunoperoxidase staining, sections (5 and 20 µm thick) were incubated in PBS containing 3% H_2_O_2_ and 20% ethanol for 5 min, to inhibit endogenous peroxidases. Sections were permeabilized and non specific epitopes were blocked by incubation for 45 min in PBS containing: 2% BSA, 0.1% Triton X-100 (tyrosine-hydroxylase (TH)-staining); 2% NGS, 5% BSA, 0.1% Triton X-100 (P^S129^-α-synuclein staining); or 20% newborn goat serum, 10% BSA, 0.1% Triton X-100 (ubiquitin staining and double immunofluorescent labelling). Sections were hybridized overnight at 4°C with the primary antibodies, then treated with biotinylated secondary anti-rabbit IgG antibody (Vectastain; Vector Laboratories, Burlingame, CA, USA), and incubated with avidin and biotinylated horseradish peroxidase complex. The signal was revealed by incubating the sections in 0.25 M Tris-HCl, pH 7.4 containing 0.5% of the peroxidase substrate, 3,3′-diaminobenzidine tetrahydrochloride, and 0.015% H_2_O_2_. The sections were dehydrated in an ascending series of ethanol concentrations, cleared in xylene, and mounted with Eukitt Mounting Medium.

For immunofluorescent staining, the sections were then incubated with the primary antibody overnight, at 4°C and the signal revealed by incubation with Alexa Fluor 488-coupled goat anti-rabbit IgG antibody (1∶2000, Invitrogen) or Cy3-coupled donkey anti-mouse IgG antibody (1∶200, Jackson), for one hour at room temperature. The same procedure was used twice sequentially for double stainings. Fluorescent images were acquired with an epifluorescensce microscope (Axioplan 2, Zeiss, Germany) equipped with the FluoUp image analysis system (Explora Nova, La Rochelle, France).

Primary antibodies were: rabbit polyclonal anti-P^S129^-α-synuclein (1∶500, kindly provided by T. Iwatsubo [1]); anti-ubiquitin (1∶1500, DAKO); anti-synphilin-1 (1∶150, kindly provided by Simone Engelender); and anti-activated caspase 9 (1∶200, Cell Signalling); rabbit monoclonal anti-P^S129^-α-synuclein (1∶5000, Abcam); mouse monoclonal anti-P^S129^-α-synuclein (1∶1500, WAKO); and anti-ubiquitin (1∶500, Chemicon). Anti-P^S87^-α-synuclein antibodies were raised in rabbits against a peptide identical to residues 81–93 of human α-synuclein, with serine 87 being phosphorylated (Eurogentec), and used at a 1∶100 dilution.

Immunostainings for total human α-synuclein and TH were done as described in Materials and Methods S1.

### Quantitative immunohistochemical analyses

The density of P^S129^-α-synuclein immunostaining was evaluated in symptomatic mice (n = 5 per genotype) by determining the mean of eight 5 µm-thick coronal hemi-brain, blind-coded sections, 120 µm apart, covering a brainstem region between bregma −2.5 and −3.5 mm, with a 25×objective and using the Mercator workstation. The borders of the brainstem area on each section were drawn manually and a grid with counting frames of 150×150 µm was superimposed onto the region of interest. The number of intersections between immunolabelled structures and grid lines was registered for each section. The mean staining density was defined as the average number of intersections per section. TH-positive neurons in the *substantia nigra* and the *locus coeruleus* were quantified as described in Materials and Methods S1 and in Reference S1.

Colocalization between P^S129^-α-synuclein and ubiquitin was analyzed in spinal cord sections processed for double immunofluorescent staining. Images of at least five double-immunostained neurites and five cell bodies from three animals per genotype were taken with a confocal microscope (Leica SP2 AOBS). Colocalization was evaluated with the Pearson's coefficient calculated with the “Colocalization Threshold” plugin of the Java image processing program Image J (http://rsb.info.nih.gov/ij/). The proportion of P^S129^-α-synuclein-positive cell bodies and neurites immunopositive for ubiquitin was determined from one or two representative double immunofluorescence-labelled, 5 µm-thick brainstem sections from symptomatic mice, chosen between bregma −2.5 and −3.5 mm according to the abundance of immunolabelled structures. To determine the proportion of P^S129^-α-synuclein-positive neurites co-stained with anti-ubiquitin antibodies, fluorescent images were acquired with an epifuorescence microscope (Axioplan 2, Zeiss, Germany), with a filter set for Alexa Fluor 488 (P^S129^-α-synuclein) and a filter set for Cy3 (ubiquitin), and a 63×objective; 30 to 130 P^S129^-α-synuclein-positive neurites were scored per animal (n = 5 per genotype). To determine the proportion of double-labelled cell bodies, brain sections were examined directly with the epifluorescent microscope and the 63×objective; 30 to 200 P^S129^-α-synuclein-positive cell bodies were scored per animal (n = 4 per genotype).

The percentage of P^S129^-α-synuclein- or ubiquitin-positive neurons double stained for cleaved caspase 9, and the percentage of neurons accumulating cleaved caspase 9 co-labelled for P^S129^-α-synuclein or ubiquitin was estimated throughout the spinal cord of symptomatic mice. Sections were examined directly with the epifluorescent microscope and the 63×objective; 140 to 660 neuronal cell bodies with P^S129^-α-synuclein, and 30 to 330 with ubiquitin staining were scored per animal (n = 4 per genotype). Seventy neurons with cleaved caspase 9 staining were scored for hA30Pα-syn mice without Parkin (n = 4), and 150 for hA30Pα-syn mice with Parkin (n = 4).

### Statistical analyses

Values from behavioural studies were analyzed using second-order polynomial curve fitting of mean performance against age, and ANOVA comparison of the corresponding regression curve parameters (slope and curvature), with genotype as qualitative factor. The regression equations were:
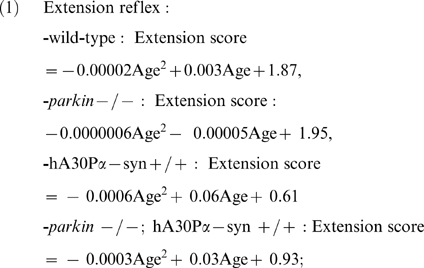


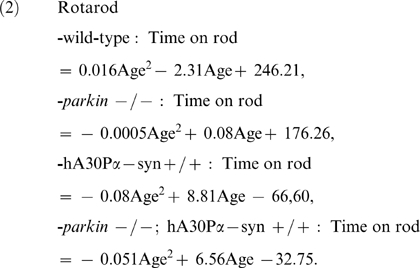



Unpaired t-tests were used to estimate differences in disease progression between groups. Data from quantitative western blots were analyzed using two-way ANOVA, followed by the Student-Newman-Keuls Method for pairwise multiple comparisons, as appropriate. An unpaired two-tailed t-test was used to estimate differences in the percentages of P^S129^-α-synuclein- or ubiquitin-positive neurons with cleaved caspase 9 staining between symptomatic hA30Pα-syn littermate mice with and without Parkin. An unpaired one-tailed t-test was used to analyse the proportion of P^S129^-α-synuclein-positive structures co-labelled with anti-ubiquitin antibodies.

## Supporting Information

Results S1 (0.03 MB DOC)

Materials and Methods S1 (0.03 MB DOC)

Reference S1 (0.02 MB DOC)

Table S1 (0.03 MB DOC)

Figure S1 Thy-1-driven expression of the hA30PÎ±-syn transgene throughout the brain and spinal cord of aged mice. A, Representative micrographs showing immunohistochemical labelling of human Î±-synuclein in various brain and spinal cord regions from healthy hA30PÎ±-syn 17 months-old mice (a–f), and from end-stage symptomatic mice presenting pathological profiles (arrows) (g–i). Both typical, dotted synaptic labelling and staining of the somatodendritic compartment were observed. Similar results were obtained in the presence or absence of Parkin. Scale bar: 100 Î¼m. B, Î±-Synuclein immunoreactivity is absent from the cell bodies of monoaminergic neurons of the substantia nigra and the locus coeruleus. Representative micrographs of confocal laser-scanned brain sections from 17 months-old hA30PÎ±-syn mice, immunolabelled by double fluorescence with anti-human Î±-synuclein and anti-TH antibodies. Arrows and arrowheads indicate TH- and human Î±-synuclein-immunopositive neurons, respectively. Similar results were obtained in the presence and absence of Parkin. Scale bar: 50 Âμm.
(11.19 MB TIF)

Figure S2 Parkin deficiency does not affect the survival of the dopaminergic neurons of the SNc in 17 months-old hA30PÎ±-syn mice. A, Micrographs illustrating TH-immunoreactivity in the substantia nigra of mice representative of each genotype. B, Stereological quantification of TH-positive neurons in the substantia nigra pars compacta (n = 4). Data presented are means±SEM. Scale bar: 100 Âμm.
(6.69 MB TIF)
