# Supplementary material for: Parkin Deficiency Delays Motor Decline and Disease Manifestation in a Mouse Model of Synucleinopathy
Source: PLoS One. 2009 Aug 14;4(8):e6629. doi: 10.1371/journal.pone.0006629 (PMC2722082; doi:10.1371/journal.pone.0006629)
Supplement: Results S1 — (0.03 MB DOC) [file pone.0006629.s001.doc]

**Supporting Results**

**Parkin deficiency does not alter the regional and subcellular distribution of total human α-synuclein in a double transgenic mouse model.**

We investigated whether the absence of Parkin affected the regional distribution or neuronal accumulation of transgenic α-synuclein in aged healthy mice, and in symptomatic mice at disease end-stage. Brain and spinal cord sections from animals of each genotype were analyzed by immunohistochemistry using antibodies specifically recognizing human α-synuclein. As previously reported, immunoreactivity to human α-synuclein was observed in various regions throughout the brain and spinal cord of single transgenic hA30Pα-syn mice (Figure S1A a-f). A synaptic distribution of the human protein, typical of that reported for endogenous murine α-synuclein [37, 53], was observed in many brain regions; in addition, and as expected, transgenic α-synuclein accumulated abnormally and massively throughout the somato-dendritic compartment of many neurons. These regional and subcellular patterns of distribution of transgenic α-synuclein were similar in the presence and absence of Parkin. In symptomatic mice of both genotypes, pathologically swollen, spherical or elongated neuritic profiles appeared in the spinal cord and in particular brain regions, including the *substantia nigra* *pars reticulata* and the pontine nuclei (Figure S1A g-i).

To determine whether the human α-synuclein transgene was expressed in brainstem catecholaminergic neurons, sections through the *substantia nigra* and *locus* *coeruleus* were probed with antibodies against TH and human α-synuclein (Figure S1B). Immunoreactivity to human α-synuclein was not detected within any of the TH-positive neurons themselves, whether or not Parkin was present, indicating the transgene expression in these neurons is low or absent.

**Parkin deficiency in hA30Pα-syn transgenic mice does not affect survival of catecholaminergic neurons in the brainstem or neurotransmitter metabolism in the corresponding projection regions.**

Consistent with the lack of human α-synuclein immunoreactivity in TH-positive neurons, there were no significant differences in their numbers in the presence or absence of Parkin. We counted TH-immunoreactive neurons in the *substantia nigra pars compacta*  (Figure S2) and the *locus coeruleus* (not shown) of 17 months-old mice. The distribution and intensity of TH-immunolabelling and the numbers of TH-positive neurons were similar in single transgenic, double transgenic and wild-type mice in both regions. In addition, there were no significant differences in dopamine metabolism between groups, as revealed by striatal dopamine, 3,4-dyhydroxyphenyl acetic acid, homovanillic acid and 3-methoxytyramine levels (Table S). Noradrenaline contents were also similar in the olfactory bulb and in the spinal cord (not shown).
