# Supplementary material for: Parkin Deficiency Delays Motor Decline and Disease Manifestation in a Mouse Model of Synucleinopathy
Source: PLoS One. 2009 Aug 14;4(8):e6629. doi: 10.1371/journal.pone.0006629 (PMC2722082; doi:10.1371/journal.pone.0006629)
Supplement: Materials and Methods S1 — (0.03 MB DOC) [file pone.0006629.s002.doc]

**Supporting Materials and Methods**

**Immunohistochemistry.**

Tissue processing and staining was as described in the Materials and Methods section. Primary antibodies were: rabbit polyclonal anti-tyrosine hydroxylase (anti-TH, 1:1000, Pel Freeze, Rogers, AR, USA); rat monoclonal anti-human α-synuclein 15G7 (1:200, [14]); and mouse monoclonal anti-human α-synuclein LB509 (1:50, Zymed). Colocalization of α-synuclein and TH was examined with a confocal laser-scanning microscope (Leica, SP2 AOBS).

**Quantitative analysis of the number of TH-positive neurons.**

Right-half brains (hemi brains) were cryoprotected by incubation for 48 hours in PBS containing 30% sucrose, then frozen in isopentane at –30°C and stored at –80°C. Free-floating cryomicrotome-cut sections (20 µm thick) from the SN and the LC were kept at 4°C in PBS with 0.02% sodium azide. Immunoperoxidase staining for TH was done as described in the Materials and Methods section. For quantitative analysis of the number of TH-positive cells, the *substantia nigra pars compacta* was defined according to Nelson et al. [Supporting Reference]; cells were counted on every 10th coronal, blind-coded hemi-brain section throughout the rostrocaudal axis of the SN from 4 female mice per genotype, using a microscope (Leitz) equipped with a 40 x objective (PL FLUOTAR, Leitz Wetzlar, numerical aperture 0.6). A Mercator digital imaging workstation (Explora Nova, La Rochelle, France) and the unbiased stereological optical fractionator method were used for quantification. Differences in the number of TH-positive neurons in the *substantia nigra* and the *locus coeurleus* were estimated by one-way ANOVA.

**HPLC analysis of tissue cathecolamines and metabolites.**

At 17 months of age, littermate mice (females) of each genotype were decapitated, then brains and spinal cords were rapidly removed and the regions of interest dissected. Freshly dissected hemi-brain regions and lumbar spinal cords were weighed, collected in 125 µl of ice-cold extraction-buffer (0.1 M HClO_4_, 0.05% EDTA, 0.05% Na_2_SO_5_) and homogenized by sonication. Homogenates were centrifuged (30,000 x g, 20 min, 4°C), and the supernatants stored at - 80°C. On day of the assays, the supernatants were thawed on ice and neutralized with 2 M K_2_HPO_4_/KH_2_PO_4_ (pH 7.0) supplemented with ascorbate oxidase (10 µg/ml; Boehringer Mannheim) and centrifuged (30,000 x g, 10 min, 4°C). Aliquots of the supernatants (10 µl) were injected onto a column (Ultrasphere IP, 25 cm, 0.46 cm OD, 5 µm; Beckman Coulter). Eluted neurotransmitters and metabolites were quantified electrochemically at 100 nA (Coulochem: DC2, Timeline 2, mobile phase : 70 mM KH_2_PO_4_, 2 mM triethylamine, 0.1 mM EDTA, 16 % methanol, 1.25 mM octane sulphonate, pH 2.78 adjusted with solid citric acid; elution rate: 1 ml/min).

Data were analysed by one-way ANOVA with the mixed procedure of SAS software.
