# Supplementary material for: Parkin Deficiency Delays Motor Decline and Disease Manifestation in a Mouse Model of Synucleinopathy
Source: PLoS One. 2009 Aug 14;4(8):e6629. doi: 10.1371/journal.pone.0006629 (PMC2722082; doi:10.1371/journal.pone.0006629)
Supplement: Reference S1 — (0.02 MB DOC) [file pone.0006629.s003.doc]

**Supporting Reference**

# Nelson EL, Liang CL, Sinton CM, German DC (1996) Midbrain dopaminergic neurons in the mouse: computer-assisted mapping. J Comp Neurol 369: 361-371.
