# Supplementary material for: Parkin Deficiency Delays Motor Decline and Disease Manifestation in a Mouse Model of Synucleinopathy
Source: PLoS One. 2009 Aug 14;4(8):e6629. doi: 10.1371/journal.pone.0006629 (PMC2722082; doi:10.1371/journal.pone.0006629)
Supplement: Table S1 — (0.03 MB DOC) [file pone.0006629.s004.doc]

**Table S1**

**Parkin deficiency does not affect the concentrations of dopamine or its metabolites in the *striatum* of hA30Pα-syn mice**

| ng/mg  genotype | DA | 3MT | HVA | Dopac |
| --- | --- | --- | --- | --- |
| Wild-type | 11.88 ± 0.31 | 1.08 ± 0.08 | 1.41 ± 0.15 | 1.66 ± 0.08 |
| *parkin* -/- | 11.26 ± 1.17 | 1.04 ± 0.04 | 1.17 ± 0.12 | 1.43 ± 0.16 |
| *parkin* -/-; hA30Pα-syn +/+ | 10.70 ± 1.98 | 1.08 ± 0.03 | 1.34 ± 0.31 | 1.40 ± 0.12 |
| hA30Pα-syn +/+ | 10.6 ± 1.45 | 1.06 ± 0.04 | 1.17 ± 0.14 | 1.71 ± 0.14 |

HPLC analysis of tissue concentrations of dopamine and its metabolites in 17 months-old female mice (n=3-5). Values reported are means ± SEM expressed as ng per mg of fresh tissue. Statistical analysis did not reveal any significant differences between groups. DA: dopamine; 3MT: 3-methoxytyramine; HVA: homovanillic acid; Dopac: 3,4-dihydroxyphenyl acetic acid.

# 
