# Supplementary figures and images for: Parkin Deficiency Delays Motor Decline and Disease Manifestation in a Mouse Model of Synucleinopathy
Source: PLoS One. 2009 Aug 14;4(8):e6629. doi: 10.1371/journal.pone.0006629 (PMC2722082; doi:10.1371/journal.pone.0006629)

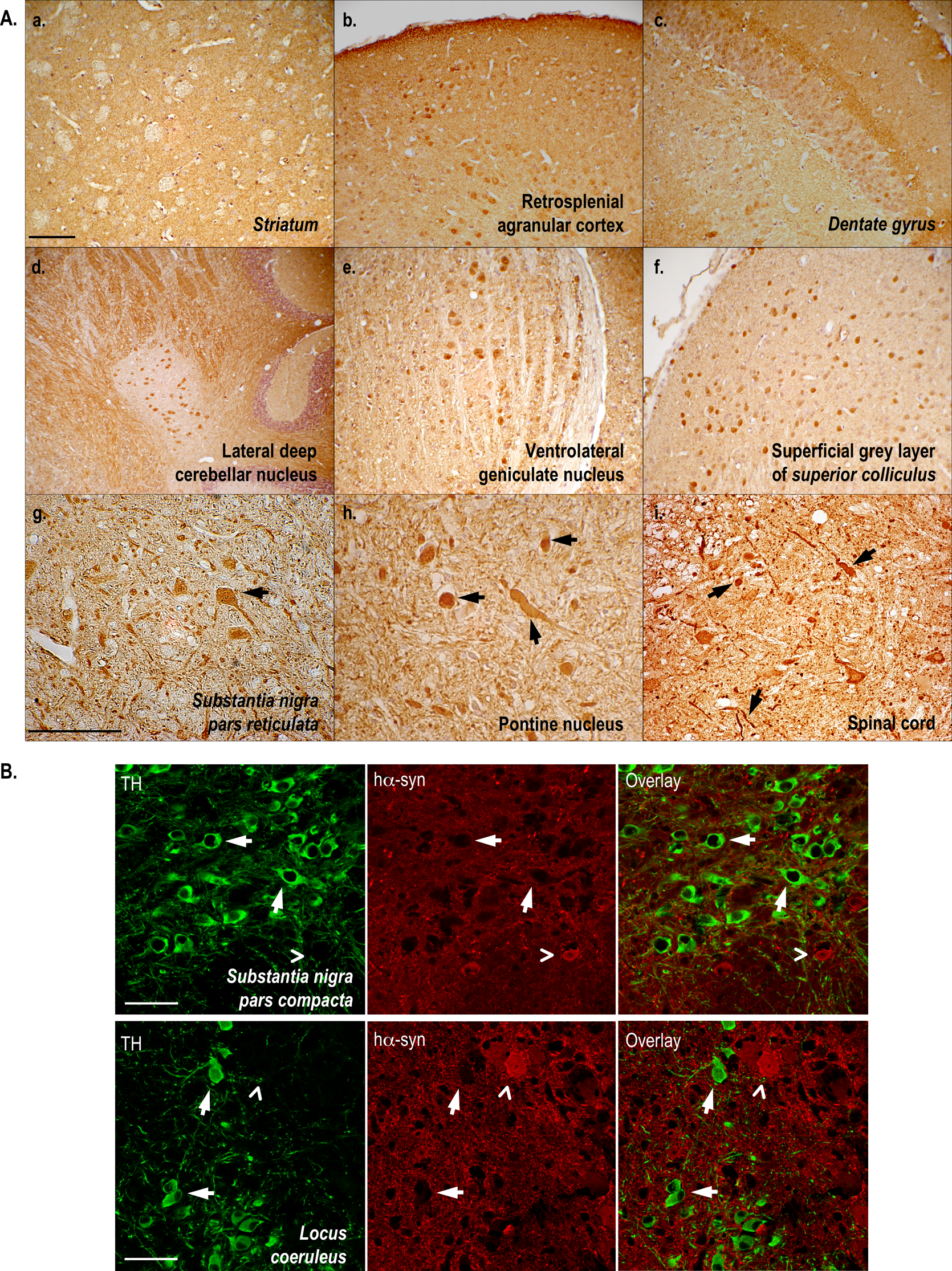

Supplement: Figure S1 — Thy-1-driven expression of the hA30PÎ±-syn transgene throughout the brain and spinal cord of aged mice. A, Representative micrographs showing immunohistochemical labelling of human Î±-synuclein in various brain and spinal cord regions from healthy hA30PÎ±-syn 17 months-old mice (a–f), and from end-stage symptomatic mice presenting pathological profiles (arrows) (g–i). Both typical, dotted synaptic labelling and staining of the somatodendritic compartment were observed. Similar results were obtained in the presence or absence of Parkin. Scale bar: 100 Î¼m. B, Î±-Synuclein immunoreactivity is absent from the cell bodies of monoaminergic neurons of the substantia nigra and the locus coeruleus. Representative micrographs of confocal laser-scanned brain sections from 17 months-old hA30PÎ±-syn mice, immunolabelled by double fluorescence with anti-human Î±-synuclein and anti-TH antibodies. Arrows and arrowheads indicate TH- and human Î±-synuclein-immunopositive neurons, respectively. Similar results were obtained in the presence and absence of Parkin. Scale bar: 50 Âμm. (11.19 MB TIF) [file pone.0006629.s005.tif]

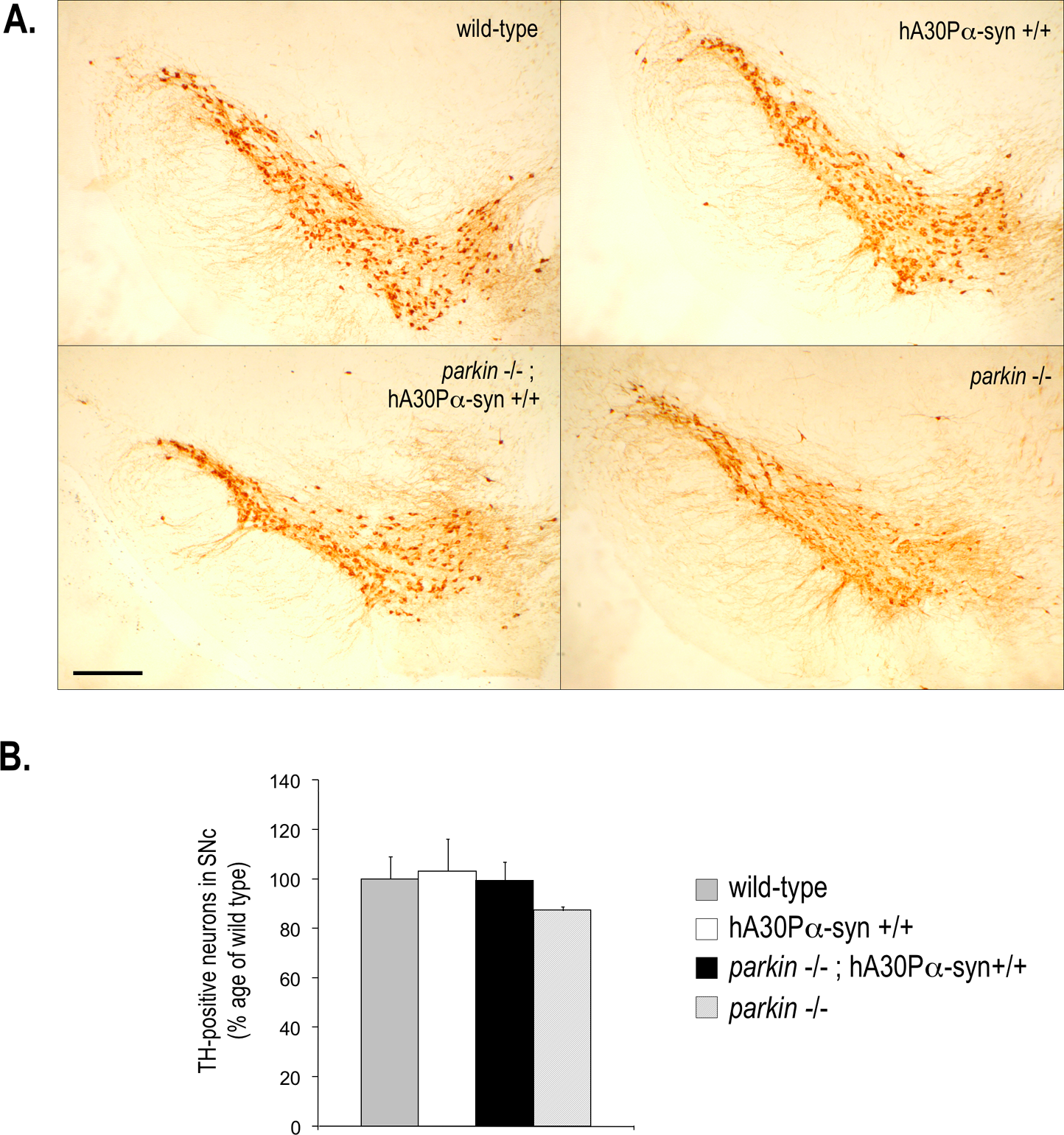

Supplement: Figure S2 — Parkin deficiency does not affect the survival of the dopaminergic neurons of the SNc in 17 months-old hA30PÎ±-syn mice. A, Micrographs illustrating TH-immunoreactivity in the substantia nigra of mice representative of each genotype. B, Stereological quantification of TH-positive neurons in the substantia nigra pars compacta (n = 4). Data presented are means±SEM. Scale bar: 100 Âμm. (6.69 MB TIF) [file pone.0006629.s006.tif]
